# Characteristics of Surgical Necrotizing Enterocolitis: Is It Different from Medical Necrotizing Enterocolitis? A Single-Center Retrospective Study

**DOI:** 10.3390/children8121148

**Published:** 2021-12-06

**Authors:** Ara Cho, Dayoung Ko, JoongKee Youn, Hee-Beom Yang, Hyun-Young Kim

**Affiliations:** 1 Department of Surgery, Division of Pediatric Surgery, Seoul National University Hospital, 101 Daehakro, Chongno-gu, Seoul 03080, Korea; ara501616@gmail.com (A.C.); kodayoung@gmail.com (D.K.); jkyoun@gmail.com (J.Y.); 2 Department of Surgery, Division of Pediatric Surgery, Seoul National University Bundang Hospital, 82, Gumi-ro 173 Beon-gil, Bundang-gu, Seongnam-si 13620, Korea; eeulere@naver.com

**Keywords:** NEC, surgical, medical, risk factor, outcome, neonate

## Abstract

(1) Background: Necrotizing enterocolitis (NEC) is one of the leading causes of death in newborns despite improvements in the care of critically ill neonates. Approximately 50–70% of the cases are managed by medical therapy. However, the remaining patients require surgical intervention. The purpose of our study was to analyze the factors associated with patients requiring surgical treatment compared to patients requiring only medical treatment; (2) Method: Patients diagnosed with necrotizing enterocolitis over a period of 14 years (January 2003–December 2016) in a single tertiary referral children’s hospital were retrospectively enrolled. Demographics and clinical data were collected through the medical record and were analyzed using Pearson’s χ^2^ test, t-tests, and linear regression; (3) Results: A total of 189 NEC patients were analyzed. In the surgical NEC group, gestational age was lower (*p* = 0.018), body weight at birth was lower (*p* = 0.034), comorbidity with respiratory distress syndrome (RDS) was higher (*p* = 0.005), the days of antibiotic use were greater (*p* = 0.014), the percentage of breast milk feeding was lower (*p* = 0.001), and the length of hospital stay was longer (*p* < 0.000). The in-hospital mortality between the two groups was not significantly different (*p* = 0.196). In multivariate logistic analysis, breast milk feeding remained less associated with surgical NEC (OR = 0.366, 95% CI: 0.164–0.817), whereas the length of hospital stay was more associated with surgical NEC (OR = 1.010, 95% CI: 1.001–1.019); (4) Conclusion: Comparing medical and surgical NEC, a significantly lower percentage of surgical NEC patients were fed breast milk and their hospital stays were longer.

## 1. Introduction

NEC is characterized by intestinal necrosis in the ileum, jejunum, and colon and is one of the major causes of morbidity and mortality in premature infants [1]. Occurring in 0.5 to 5 per 1000 live births, NEC is more common in low-weight infants [2,3]. The pathophysiology of NEC is not fully understood, but several risk factors have been reported. Immaturity, ischemia, altered gut microflora, decreased mucin barrier, increased gut permeability, decreased gut immunity, and certain types of enteral feeding formulas have been reported as risk factors for NEC [4,5,6,7]. Several of the identified risk factors result in oxidative stress, inflammation, and ultimately necrosis of the neonatal bowel [4,5].

NEC is diagnosed by a combination of clinical symptoms and signs, including feeding intolerance, abdominal distension, and bloody stools. Radiologic features include pneumatosis intestinalis, portal venous gas, and pneumoperitoneum [8].

When NEC is diagnosed or strongly suspected, medical management begins. Medical management should stop all enteral feeding, perform gastric decompression via orogastric tubes, inject intravenous fluids, inject intravenous broad-spectrum antibiotics, and ensure central intravenous access [9]. Surgical intervention, which consists of removing necrotic intestine and performing an ileostomy or anastomosis, is required in 30–50% of all NEC cases [10].

There is still insufficient information on whether the clinical characteristics of surgical and medical NEC patients differ. Thus, the purpose of this study was to compare medical and surgical NEC patients to identify clinical factors that identify which NEC patients will require surgical intervention.

## 2. Materials and Methods

### 2.1. Study Design

This study was a retrospective study whose protocol was approved by the institutional review board of the Seoul National University Hospital (SNUH) (IRB number: 1911-006-1074). The informed consent was waived because of the retrospective design of the study. We retrospectively evaluated patients with NEC over a period of 14 years (March 2003–September 2016) at the Seoul National University Children’s Hospital (SNUCH). The diagnosis of NEC was done in reference to the modified Bell staging [11]. This study enrolled stage I, II, and III of enterocolitis. Stage I was suspected and unproven enterocolitis, most of these children did not progress to stage II or III. Stage II was definite enterocolitis which included pneumatosis intestinalis, ascites, and tenderness of the abdomen. Patients in stage I and II were medically treated. Stage III was enterocolitis with signs of peritonitis and intestinal perforation. Patients in stage III were treated surgically. Patients diagnosed with NEC who did not receive surgical therapy were defined as medical NEC patients, and patients who received surgical treatment were defined as surgical NEC patients.

### 2.2. Variables

Data on the factors related to patient demographics, maternal factors, clinical factors, laboratory, and treatment factors were recorded. The demographics included sex, gestational age (GA) (weeks), body weight at birth (g), body weight at birth (extremely low body weight [ELBW] less than 1000 g, very low body weight [VLBW] between 1000 g and 1500 g), location of the birth (hospital in-born or out-born), the method of delivery (vaginal delivery or cesarean section), small size for gestational age, multiple births, Apgar score at one minute and five minutes, cord pH, body temperature at neonatal intensive care unit (NICU) admission, comorbidities (respiratory distress syndrome (RDS), moderate to severe bronchopulmonary dysplasia (BPD), retinopathy of prematurity (ROP) surgery, intraventricular hemorrhage (IVH) grade > 3, periventricular leukomalacia (PVL), medical patent ductus arteriosus (PDA), and surgical patent ductus arteriosus), associated gastrointestinal (GI) anomalies, and associated cardiac anomalies (except PDA, patent foramen ovale (PFO), or arterial switch operation (ASO)). Maternal factors included: maternal age (years), maternal diabetes mellitus (DM), hypertension (HTN), history of maternal steroid usage, polyhydramnios, oligohydramnios, placental abruption, cause of prematurity (spontaneous labor, premature rupture of membrane (PROM), fetal distress, and other causes), and the number of days of antibiotic use for PROM. Laboratory and treatment factors included: antibiotic use, transfusions, exchange transfusions, indomethacin or ibuprofen use, postnatal steroid use, sepsis, number of days of antibiotic use (days), highest WBC count, lowest hemoglobin level, lowest platelet count, highest C-reactive protein (CRP) level, day of first feed, day first full feeding accomplished, type of material feed (breast milk or other formula), time of meconium pass (hours), length of hospital stay (days), and in-hospital mortality. All patients were followed-up until hospital discharge or death.

### 2.3. Statistical Analysis

To evaluate categorical variables, we used χ^2^ tests or Fisher’s exact test. To compare continuous variables, we used Student’s t-test or the Mann-Whitney U-test. Factors associated with surgical NEC were identified by univariate analysis and their corresponding *p*-values. Variables with *p*-values < 0.05 were included in the multivariate analysis. Based on the logistic regression model, correlations between the identified factors and surgical NEC were analyzed. Statistical analysis was performed using the Statistical Package for Social Science (SPSS) version 25.0.

## 3. Results

### 3.1. Patients Demographics

Of the 189 patients, 102 were treated with medical intervention and 98 patients were treated with surgical intervention. The two groups are compared in Table 1. The proportion of male patients was 57.5% in the surgical NEC group and 31.7% in the medical NEC group. The average gestational age was 26.5 ± 2.5 weeks in the surgical NEC group and 27.4 ± 2.4 weeks in the medical NEC group (*p* = 0.018). The average body weight at birth was 821.5 ± 260.2 g in the surgical NEC patients and 888.5 ± 227.3 g in the medical NEC patients (*p* = 0.034). The percentage of patients who were small for gestational age was 36.1% in the surgical NEC group and 26.7% in the medical NEC group. RDS is a disorder primarily caused by the deficiency of pulmonary surfactant in immature lung. RDS was diagnosed based on a clinical manifestation of respiratory failure and characteristic radiologic findings. RDS was seen in 82.6% of the surgical NEC patients and 64.3% of the medical NEC patients (*p* = 0.005). 

### 3.2. Maternal Factors, Laboratory Factors and Treatment Factors of Patients

Maternal factors of the NEC patients are listed in Table 2. Laboratory and treatment factors are shown in Table 3. More surgical NEC patients were treated with antibiotics (94.0%) than medical NEC patients (85.9%). The number of antibiotic use days was 30.4 ± 27.4 in the surgical NEC patients and 20.9 ± 21.9 in the medical NEC patients (*p* = 0.014). The highest WBC count was 21.9 ± 14.2 × 103/μL in the surgical NEC patients and 21.1 ± 13.0 × 103/μL in the medical NEC patients. The highest CRP level was 11.5 ± 48.6 mg/dL in the surgical NEC patients and 4.5 ± 5.1 mg/dL in the medical NEC patients. Exclusive breast feeding occurred in 29.5% of the surgical NEC patients, while the rest were either fed formula or a mixture of the two (70.5%). This is in contrast to medical NEC patients, where 57.6% were exclusively breast fed, and 42.4% were fed formula or a mixture of the two (*p* = 0.001). The length of hospital stay was 99.7 ± 61.5 days for the surgical NEC patients and 73.5 ± 37.4 in the medical NEC patients (*p* = 0.000). The in-hospital mortality rate was 16.1% in the surgical NEC patients and 9.8% in the medical NEC patients.

### 3.3. Risk Factors of Surgical NEC

In a multivariate analysis of the factors associated with surgical NEC, six factors with *p*-values < 0.05 were analyzed (Table 4). Surgical NEC patients had reduced odds of being exclusively breast fed than medical NEC patients (OR 0.366; 95% CI: 0.164–0.817). Surgical NEC patients also had increased odds of a longer hospital stay compared to medical NEC patients (OR 1.010; 95% CI: 1.001–1.019).

## 4. Discussion

Early predictors of progression to surgical NEC are of great interest in NEC research [12]. In the absence of a clear pneumoperitoneum, signs in physical examinations, such as a fixed abdominal mass and abdominal wall erythema, are the best evidence of intestinal necrosis [13]. According to Kosloske’s retrospective cohort study of 147 NEC patients, pneumoperitoneum, portal venous gas, and positive paracentesis were the best indicators of surgical intervention, with a positive predictive value of 100% and a prevalence of 410%. Positive paracentesis refers to positive bacteria in brown fluid or gram stains. However, there is controversy over the indications for paracentesis. Kosloske recommends paracentesis in cases of clinical deterioration despite medical management or with extensive pneumatosis intestinalis [14]. Kosloske’s recommendation of portal venous gas as a predictor of surgical intervention has been questioned by other retrospective reviews that have reported a low value for portal venous gas in predicting NEC. Sharma et al. reported that about half of the NEC patients with portal venous gas survived without surgery [14,15,16].

Tepas et al. reported a predictive model including seven metabolic derangements. Seven parameters (positive blood culture, acidosis, bandemia, thrombocytopenia, hyponatremia, hypotension, and neutropenia) were scored in a binary manner to assess disease severity and determine the need for surgical intervention [17]. Using this model, outcomes were improved in patients with Bell’s stage II or III NEC compared to using presence of pneumoperitoneum in decision-making [18].

Biomarker studies have been conducted to predict which patients will progress to surgical NEC. Severe thrombocytopenia (<100 × 109/L) correlated with indications of disease extension, mortality, and laparotomy [19,20,21]. Reisinger et al. reported that urinary serum amyloid A (SAA) levels and platelet counts could be used to predict surgical NEC accurately, with sensitivities of 94% and specificities of 83% [22]. Fecal calprotectin (S100A8/S100A9 heterodimer) distinguishes between Bell’s stage II and Bell’s stage III with 76% sensitivity and 92% specificity. Another fecal protein, S100A12, was higher in the bowel perforation group of patients with suspected NEC than in the other group [23]. Some studies have shown that elevated CRP implies an advanced stage of NEC, [24] while other studies have reported no association with disease severity. Significantly higher CRP levels were reported in surgical NEC patients compared to medical NEC patients (2625 pg/mL vs. 156 pg/mL, respectively, *p* < 0.001) [25].

Miriam et al. compared 170 patients in Italy and found that surgical NEC patients had lower GA (*p* < 0.0001), lower birth weight (*p* = 0.0006), and lower onset body weight (*p* < 0.0001) compared to medical NEC patients. There were also more ELBW patients (*p* = 0.0033), more PDA (*p* = 0.0033), more use of NSAIDs (*p* = 0.0229), earlier onset of NEC (*p* = 0.0003), more days to reach full enteral feeding (*p* < 0.0001), longer hospital stays (*p* < 0.0001), higher mortality (*p* = 0.0003), and higher platelet counts (*p* = 0.0071) in the surgical NEC patients. In multivariate regression analysis, later onset of NEC, higher pH values, and intestinal pneumatosis on abdominal x-ray were associated with a lower risk of surgery, whereas higher CRP levels at NEC onset were associated with a higher risk of surgery [26]. 

Similar to the results of the Miriam study, we found that surgical NEC patients had a lower GA and lower birth weight and longer hospital stay, but we did not find significant correlations with the proportion of ELBW patients, PDA, days to reach full feeding, and hospital mortality. Regarding laboratory factors, we did not find that WBC, hemoglobin levels, platelet counts, or CRP levels were associated with surgical NEC. Further prospective studies are required to collect information on new factors and to corroborate those factors found to be important in previous studies.

A strength of this study was that we identified formula and mixed feeding as a significant risk factor for surgical NEC. A previous study of patients in the early postnatal period reported that the components of breast milk were protective against NEC, not only in exclusive feeding but also in partial feeding, with a dose-dependent relationship between exclusively of breast milk feeding and its protective effect [26]. Breast milk contains both nutrient and non-nutrient components. The non-nutrient components include immunoglobulins and lactoferrin, which aid in intestinal adaptation, maturation, food tolerance, and protection against infective and inflammatory disorders [27]. The absence of protective proteins in breast milk and the presence of foreign bovine proteins can be detrimental to intestinal immune balance. It can also drive microbiota dysbiosis, which is a disturbance in the composition of the microbiota in the intestine. The impact of probiotics and breast milk in NEC patients indicates that intestinal dysbiosis is an important mechanism in the progression of NEC [28]. It is recommended that in preterm babies with known risk factors of NEC, it would be helpful to have human milk feeding in advance; because it has protective effect. In addition to the importance of breast milk, they reported on enteral feeding strategies and microbiota analysis. Slower and intermediate rates of progression of enteral feeding strategies were associated with a higher risk of NEC. Also, less favorable and intermediate direct-breastfeeding policies were associated with higher risk of NEC. Microbiota analysis resulted in the association between *Clostridium neonatale* and *Staphylococcus aureus* with NEC. This report implies that feeding strategies and microbiota as well as feeding materials are important and should be considered in future studies [29]. 

There were some limitations to this study. First, there are no definite unit protocols on trophic feeding in NEC patients. However, the doctor determines the feeding schedule by comprehensively considering the physical exam, radiologic findings, and laboratory results. In case of suspected peritonitis or perforation, trophic feeding is delayed. Elsewhere, early trophic feeding is recommended for nutrition and growth.

This study is retrospective in nature and data was collected retrospectively. Some detailed data were not collected, such as the timing of antibiotic usage and the presence of chorioamnionitis in the mother. Also, there is a lack of data on physical examinations and radiologic findings associated with surgical treatment decisions. Identifying significant factors by using all available evidence including imaging, clinical data, laboratory findings, and maternal factors can help improve the determination of the best parameters to predict surgical intervention. 

## 5. Conclusions

In conclusion, we found that lower levels of exclusive breast milk feeding and longer hospital stays were significantly associated with the need for surgery in NEC patients. Further multi-center studies with larger sample sizes and long-term follow-up will clarify the factors associated with the progression from medical NEC to surgical NEC. 

## Figures and Tables

**Table 1:** Patient demographics.

Characteristics	Total (N = 189)	Surgical NEC (N = 87)	Medical NEC (N = 102)	*p*-Value
Male	110 (58.2)	50 (57.5)	60 (31.7)	0.851
Gestational age (weeks)	27.0 ± 2.5	26.5 ± 2.5	27.4 ± 2.4	0.018
Body weight at birth (g)	857.7 ± 244.6	821.5 ± 260.2	888.5 ± 227.3	0.034 *
Body weight				
ELBW (<1.0 kg)	131 (69.3)	62 (71.3)	69 (67.6)	0.591
VLBW (<1.5 kg)	58 (30.7)	25 (28.7)	33 (32.4)	
Location of birth				
In-born	117 (61.6)	48 (55.2)	69 (67.6)	0.078
Out-born	72 (38.1)	39 (44.8)	33 (32.4)	
Method of delivery				
Vaginal delivery	64 (34.8)	30 (34.9)	34 (34.7)	0.978
Cesarean section	120 (65.2)	56 (65.1)	64 (65.3)	
Small for gestational age	49 (31.0)	26 (36.1)	23 (26.7)	0.205
Multiple births	90 (49.7)	43 (52.4)	47 (47.5)	0.506
Apgar score				
Apgar score at 1 min	3.5 ± 2.1	3.4 ± 2.1	3.5 ± 2.2	0.784
Apgar score at 5 min	5.7 ± 2.0	5.6 ± 2.0	5.8 ± 1.9	0.514
Cord pH	7.3 ± 0.1	7.3 ± 1.0	7.3 ± 1.0	0.373
Body temperature at NICU admission (°C)	35.8 ± 1.1	35.8 ± 1.3	35.7 ± 1.0	0.506 *
Comorbidity				
RDS	134 (72.8)	71 (82.6)	63 (64.3)	0.005
Moderate to severe BPD	86 (63.7)	43 (71.7)	43 (57.3)	0.085
ROP operation	52 (48.6)	30 (57.7)	22 (40.0)	0.067
IVH grade > 3	23 (15.4)	10 (15.2)	13 (15.7)	0.932
PVL	18 (10.2)	9 (10.8)	9 (9.7)	0.799
PDA				
Medical PDA	89 (49.2)	43 (51.2)	46 (47.4)	0.473 ***
Surgical PDA	52 (28.7)	25 (29.8)	27 (27.8)	
Associated GI anomaly	2 (1.1)	1 (1.1)	1 (1.0)	1.000 **
Associated cardiac anomaly (except PDA, PFO, ASO)	12 (6.5)	6 (7.0)	6 (6.1)	0.801

Data are presented as mean ± SD, or number (%). *p*-value: comparison of surgical and surgical NEC. * Mann-Whitney U test. ** Fisher’s exact test. *** Linear by linear association. NEC = necrotizing enterocolitis, NICU = neonatal intensive care unit, RDS = respiratory distress syndrome, BPD = bronchopulmonary dysplasia, ROP = retinopathy of prematurity, IVH = intraventricular hemorrhage, PVL = periventricular leukomalacia, PDA = patent ductus arteriosus, GI = gastrointestinal, PFO = permanent foramen ovale, ASO = arterial switch operation.

**Table 2:** Maternal factors.

Characteristics	Total (N = 189)	Surgical NEC (N = 87)	Medical NEC (N = 102)	*p*-Value
Maternal age (years)	32.8 ± 4.4	32.9 ± 5.0	32.6 ± 4.0	0.642
Maternal DM	8 (5.1)	4 (5.4)	4 (4.8)	1.000 **
Maternal HTN	24 (14.8)	8 (10.8)	16 (18.2)	0.188
Maternal steroid history	79 (54.9)	39 (58.2)	40 (51.9)	0.451
Polyhydramnios	8 (6.6)	5 (9.4)	3 (4.4)	0.296 ** 0.906
Oligohydramnios	20 (16.5)	9 (17.0)	11 (16.2)	
Placental abruption	5 (3.2)	1 (1.4)	4 (4.8)	0.372 **
Cause of prematurity				
Preterm labor with spontaneous labor	39 (24.7)	18 (24.3)	21 (25.0)	0.650 ***
Preterm labor with PROM	63 (39.3)	30 (40.5)	33 (39.3)	
Fetal distress	28 (17.7)	10 (13.5)	18 (21.4)	
Other causes	28 (17.7)	16 (21.3)	12 (14.3)	
Antibiotic use for PROM (days)	10.7 ± 14.1	13.5 ± 12.3	9.1 ± 14.9	0.301

Data are presented as mean ± SD, or number (%). *p*-value: comparison of surgical and surgical NEC. ** Fisher’s exact test. *** Linear by linear association. DM = diabetes mellitus, HTN = hypertension, PROM = premature rupture of membrane.

**Table 3:** Laboratory and treatment factors.

Characteristics	Total (N = 189)	Surgical NEC (N = 87)	Medical NEC (N = 102)	*p*-Value
Antibiotics	158 (89.8)	79 (94.0)	79 (85.9)	0.074
Transfusion	150 (84.7)	75 (90.4)	75 (79.8)	0.051
Exchange transfusion	1 (0.6)	1 (1.2)	0 (0.0)	0.468 **
Indomethacin or ibuprofen	104 (60.1)	46 (57.5)	58 (62.4)	0.515
Postnatal steroids	12 (7.2)	6 (7.9)	6 (6.6)	0.746 0.066
Sepsis	92 (52.3)	50 (59.5)	42 (45.7)	
Antibiotic usage (days)	25.5 ± 25.1	30.4 ± 27.4	20.9 ± 21.9	0.014 *
Highest WBC (x/μL)	21.5 ± 13.5	21.9 ± 14.2	21.1 ± 13.0	0.676
Lowest Hb (g/dL)	8.7 ± 2.0	8.9 ± 2.3	8.5 ± 1.6	0.231 *
Lowest Plt (x/μL)	111.0 ± 110.1	130.7 ± 146.1	93.7 ± 62.8	0.213 *
Highest CRP (mg/dL)	7.6 ± 32.7	11.5 ± 48.6	4.5 ± 5.1	0.962 *
First diet (days)	7.8 ± 8.6	7.0 ± 6.4	8.2 ± 9.8	0.423
Full feeding (days)	34.3 ± 28.3	42.7 ± 38.1	29.4 ± 18.9	0.069 *
Feeding material				
Breast milk	71(46.4)	18 (29.5)	53 (57.6)	0.001
Formula & mixed	82 (53.6)	43(70.5)	39 (42.4)	0.001
Meconium passing time (hours)	14.9 ± 16.9	15.2 ± 22.0	14.7 ± 12.2	0.878
Length of hospital stay (days)	85.4 ± 51.3	99.7 ± 61.5	73.5 ± 37.4	0.000 *
Mortality in hospital	24 (12.7)	14 (16.1)	10 (9.8)	0.196

Data are presented as mean ± SD, or number (%). *p*-value: comparison of surgical and surgical NEC. * Mann-Whitney U test. ** Fisher’s exact test. WBC = white blood cell, Hb = hemoglobin, Plt = platelet, CRP = C-reactive protein.

**Table 4:** Multivariate logistic analysis of the factors associated with surgical necrotizing enterocolitis.

	Adjusted Odds Ratio (95% Confidence Interval)	*p*-Value
Breast milk feeding	0.366 (0.164–0.817)	0.014
RDS	2.784 (0.981–7.905)	0.054
Length of hospital stay (days)	1.010 (1.001–1.019)	0.036

RDS = respiratory distress syndrome.
